# 839 Intraoperative Whole Blood Transfusion Improves Outcomes in Pediatric Burn: 1st Reported Case

**DOI:** 10.1093/jbcr/iraf019.370

**Published:** 2025-04-01

**Authors:** Pamela Decker, James Jeng, Minh-Ha Tran, Kimberly Zaky, Shiu-Yi Chen, Kamran Urgun

**Affiliations:** University of California Irvine Health Regional Burn Center; University of California Irvine; University of California Irvine; University of California Irvine; University of California Irvine Health Regional Burn Center; University of California Irvine

## Abstract

**Introduction:**

This case study explores the innovative use of whole blood transfusion intraoperatively in a pediatric burn patient, a 7-month-old male with 21% TBSA deep partial-thickness burns due to a scalding accident. The study focuses on how whole blood transfusion helped manage blood loss and maintain hemodynamic stability during surgical excision.

**Methods:**

A multidisciplinary team (pediatric intensivists, anesthesiologists, burn specialists, cardiologists) assessed the patient and identified significant potential for blood loss during surgery. They opted for intraoperative whole blood transfusion due to its ability to:

Provide comprehensive hemostatic support with red blood cells, plasma, and platelets.

Enhance oxygen delivery, aiding the healing process. Aid in volume expansion, ensuring hemodynamic stability during surgery. The patient received 10 mL/kg of whole blood intraoperatively with continuous monitoring of vitals and blood profiles. Blood bank coordination ensured proper blood handling and availability.

**Results:**

On May 2, 2024, the patient underwent excision of necrotic tissue and cadaver skin graft placement. During the procedure, 180 mL of whole blood was administered. The surgery was successful with no major complications. The patient demonstrated stable hemodynamic indicators and required no additional blood product transfusions intraoperatively. Postoperative care in the ICU showed: Hemodynamic Stability: The patient maintained stable blood pressure and heart rate. Reduced Need for Additional Blood Products: The comprehensive nature of whole blood minimized the requirement for further transfusions. Enhanced Recovery: The patient exhibited effective wound healing and showed promising signs of overall recovery. The patient was discharged on May 23, 2024, with continued follow-up plans, including physical therapy and wound care management.

**Conclusions:**

The successful use of intraoperative whole blood transfusion is the first documented infant burn case and demonstrates a promising strategy to manage significant blood loss and improve surgical outcomes. While this case serves as an example of effective care, further research is necessary to confirm the benefits of whole blood in similar pediatric surgical contexts. Establishing standardized protocols for using whole blood in pediatric burn patients is essential to enhance care and reduce risks in complex cases.

**Applicability of Research to Practice:**

Future studies should focus on evaluating post-surgical outcomes and the specific impact of whole blood use prior to large excisions in pediatric burn patients. The post operative hemodynamic stability of this infant after a large necrotic burn excision warrants further exploration of whole blood in a broader context than currently seen today.

**Funding for the Study:**

N/A

